# Colonisation of germ-free mice with probiotic lactobacilli mitigated allergic sensitisation in murine model of birch pollen allergy

**DOI:** 10.1186/2045-7022-4-S2-P26

**Published:** 2014-03-17

**Authors:** Hana Kozakova, Martin Schwarzer, Dagmar Srutkova, Tomas Hudcovic, Bozena Cukrowska

**Affiliations:** 1 Institute of Microbiology of the Czech Academy of Sciences of the Czech Republic, Doly 183, Novy Hradek, 54922, Czech Republic; 2 Institute of Microbiology of the Czech Academy of Sciences of the Czech Republic, Laboratory of Gnotobiology, Novy Hradek, Czech Republic; 3 The Children’s Memorial Health Institute, Department of Pathology, Warsaw, Poland

## Background

Allergies are becoming a serious health burden in developed countries. Increasing numbers of clinical trials and animal experiments show that probiotics are new promising tools in allergy prophylaxis. In the present work we compared anti-allergic properties of the recently described probiotic mixture of Lactobacillus casei LOCK0900, L. casei LOCK0908 and L. paracasei LOCK0919 and L. plantarum NCIMB8826 using gnotobiotic experimental model of birch pollen allergy.

## Methods

Germ-free (GF) BALB/c mice were colonized with lactobacilli mixture or with L. plantarum. Colonized mice and age-matched GF controls were repeatedly immunized with Bet v 1. Allergen-specific antibody levels, total IgA and IgE, and transforming growth factor (TGF)-beta were determined in sera. Th1/Th2 cytokine response was measured in supernatants of spleen and mesenteric lymph node (MLN) cell cultures.

## Results

Mice colonized with lactobacilli mixture showed significantly lower value of Bet v 1-specific IgG1, IgG2a and IgE and elevated levels of total IgA in sera and intestinal lavages and increase of TGF-beta compared to the GF sensitized group. We observed no such changes in L. plantarum colonized mice. Splenocytes of mice colonized with lactobacilli mixture, cultivated with Bet v 1, showed up-regulation of TGF-beta; in contrast in mice colonized with L. plantarum it was down-regulated. In MLN the colonization increased production of TGF-beta and reduced IL-5 in both groups, L. plantarum colonization increased IFN-gamma production.

## Conclusion

Colonization with lactobacilli mixture inhibited the development of allergic immune responses through induction of regulatory cytokine TGF-beta and can be thus exploited for alleviation of pollen allergies. Supported by grants 303/09/0449 of the Czech Science Foundation, CZ.3.22/2.1.00/09.01574 and WTZ CZ16 of the OEAD, NR12-0101-10/2011 of the Republic of Poland.

